# Addressing health inequity during the COVID-19 pandemic through primary health care and public health collaboration: a multiple case study analysis in eight high-income countries

**DOI:** 10.1186/s12939-023-01968-6

**Published:** 2023-08-31

**Authors:** Dorien Vanden Bossche, Q. Jane Zhao, Sara Ares-Blanco, Maria Pilar Astier Peña, Peter Decat, Naoki Kondo, Madelon Kroneman, Daisuke Nishioka, Ferdinando Petrazzuoli, Guri Rortveit, Emmily Schaubroeck, Stefanie Stark, Andrew D. Pinto, Sara Willems

**Affiliations:** 1 Department of Public Health and Primary Care, Faculty of Medicine and Health Sciences, Ghent University, Ghent, Belgium; 2 Institute of Health Policy, Management and Evaluation (IHPME), University of Toronto, Toronto, ON Canada; 3 Department of Family and Community Medicine, University of Toronto, Toronto, ON Canada; 4 St Michael’s Hospital, Toronto, ON Canada; 5 Federica Montseny Primary Care Centre, Madrid, Spain; 6 Patient Safety Working Party of semFYC (Spanish Society for Family and Community Medicine), Madrid, Spain; 7 Territorial Healthcare Quality Unit, Camp de Tarragona, Health Department Generalitat de Catalunya, Healthcare Institute of Catalonia, Tarragona, Spain; 8 Department of Social Epidemiology, University of Kyoto, Kyoto, Japan; 9 Nivel (Netherlands Institute of Health Services Research), Utrecht, the Netherlands; 10 Department of Medical Statistics, Research & Development Center, Osaka Medical and Pharmaceutical University, Osaka, Japan; 11 Department of Clinical Sciences, Centre for Primary Health Care Research, Lund University, Malmö, Sweden; 12 Department of Global Public Health and Primary Care, University of Bergen, Bergen, Norway; 13 Institute of General Practice, Friedrich-Alexander University Erlangen-Nürnberg (FAU), Erlangen, Germany

**Keywords:** Primary health care, Public health, Collaboration, Equity, Vulnerable populations, COVID-19

## Abstract

**Background:**

The COVID-19 pandemic substantially magnified the inequity gaps among vulnerable populations. Both public health (PH) and primary health care (PHC) have been crucial in addressing the challenges posed by the pandemic, especially in the area of vulnerable populations. However, little is known about the intersection between PH and PHC as a strategy to mitigate the inequity gap. This study aims to assess the collaboration between PHC and PH with a focus on addressing the health needs of vulnerable populations during the COVID-19 pandemic across jurisdictions.

**Methods:**

We analyzed and compared data from jurisdictional reports of COVID-19 pandemic responses in PHC and PH in Belgium, Canada (Ontario), Germany, Italy, Japan, the Netherlands, Norway, and Spain from 2020 to 2021.

**Results:**

Four themes emerge from the analysis: (1) the majority of the countries implemented outreach strategies targeting vulnerable groups as a means to ensure continued access to PHC; (2) digital assessment in PHC was found to be present across all the countries; (3) PHC was insufficiently represented at the decision-making level; (4) there is a lack of clear communication channels between PH and PHC in all the countries.

**Conclusions:**

This study identified opportunities for collaboration between PHC and PH to reduce inequity gaps and to improve population health, focusing on vulnerable populations. The COVID-19 response in these eight countries has demonstrated the importance of an integrated PHC system. Consequently, the development of effective strategies for responding to and planning for pandemics should take into account the social determinants of health in order to mitigate the unequal impact of COVID-19. Careful, intentional coordination between PH and PHC should be established in normal times as a basis for effective response during future public health emergencies. The pandemic has provided significant insights on how to strengthen health systems and provide universal access to healthcare by fostering stronger connections between PH and PHC.

**Supplementary Information:**

The online version contains supplementary material available at 10.1186/s12939-023-01968-6.

## Background

The COVID-19 pandemic has not only triggered a public health crisis but has also induced severe economic and social crises as a result of the measures implemented to contain the virus’s spread. The consequences of the pandemic have been unevenly distributed across economies and societies, with vulnerable populations experiencing a significant exacerbation of existing inequity gaps [1]. This situation may have negative implications for the long-term physical, socioeconomic, and mental well-being of these populations [2]. In particular, frail elderly, those with low health literacy and language barriers, people living in a precarious social context, refugees and undocumented migrants, and homeless people are at increased risk for adverse health outcomes [1–6].

These vulnerable populations represent a diverse group, but encounter a great share of disadvantages and risks, including the postponement of care due to limited access to healthcare services [7]. People could postpone COVID-19 testing in case they have symptoms, which may potentially also put their relatives and communities at risk [6]. At the beginning of the pandemic, due to high workload and physical distancing measures among others, there was a delay in the provision of ‘regular’ care, which resulted in diminished communication with vulnerable populations and inadequate treatment for patients experiencing multiple chronic medical conditions [8]. Moreover, people living in poorer socioeconomic circumstances have higher rates of comorbid chronic health problems, which renders them more susceptible to contracting infections and experiencing severe consequences of the disease compared with others [6, 9]. In addition, the measures to contain the virus’s spread limited social activities, which again induced new health problems that increased the need for care, especially for vulnerable populations [10]. This inequity and disproportionate impact, which are subjacent and present in healthcare systems, did not uniquely come to the surface due to COVID-19 but have also unfolded in other global pandemics like the HIV pandemic, SARS, H1N1 and others [11, 12].

Both public health and primary health care have been crucial in addressing the challenges posed by the COVID-19 pandemic in countries with such systems in place. Public health (PH) is defined as “the art and science of preventing disease, prolonging life and promoting health through the organized efforts of society” [13]. Its primary objective is to enhance the health of populations by maintaining individuals’ well-being, improving their health status, or preventing the deterioration of diseases. A commonly drawn distinction between PH and primary care is that primary care predominantly focuses on the individual level, while PH adopts a population perspective [14]. However, this distinction offers limited utility since populations consist of individuals, and PH interventions can also be targeted at the individual level [15]. While numerous PH activities are geared towards population-level interventions, such as health campaigns, there are also PH services tailored to individuals, including screening and vaccination. Common PH endeavors encompass the surveillance of population health, prompt response to health hazards and emergencies, health protection (e.g., addressing environmental or occupational risk factors), health promotion (including measures targeting social determinants and health inequities), as well as disease prevention (including early detection strategies).

The terms primary care (PC) and primary health care (PHC) are often used interchangeably [16]. The term PHC emerged from the 1978 Alma-Ata Declaration and encompasses not only a level of care (as PC does), but also a more comprehensive approach [16]. This approach places emphasis on universal coverage, accessibility, comprehensive care, disease prevention, health promotion, intersectoral collaboration, and engagement of both communities and individuals [17]. The Alma-Ata Declaration outlined that PHC should address the predominant health issues within a community and provide services that are promotive, preventive, curative, and rehabilitative in nature, alongside multisectoral interventions [17]. Consistent with this perspective, the World Health Organization (WHO) defines PHC as comprising three key elements: empowerment of individuals and communities, multisectoral policy and action, and the integration of PC and essential PH functions at the core of comprehensive health services [18]. The WHO further clarifies that PHC encompasses a broad range of services spanning from prevention measures, such as vaccinations and family planning, to the management of chronic health conditions and palliative care [18]. When discussing primary healthcare (PHC), it is important to note that it encompasses more than just general practitioners (GPs). The PHC system encompasses various components of the healthcare system, such as outpatient care for women’s health, children’s health, mental health, and other related services. Additionally, it may extend to include social services and community organizations, broadening its scope beyond the confines of medical care.

**Fig. 1:**
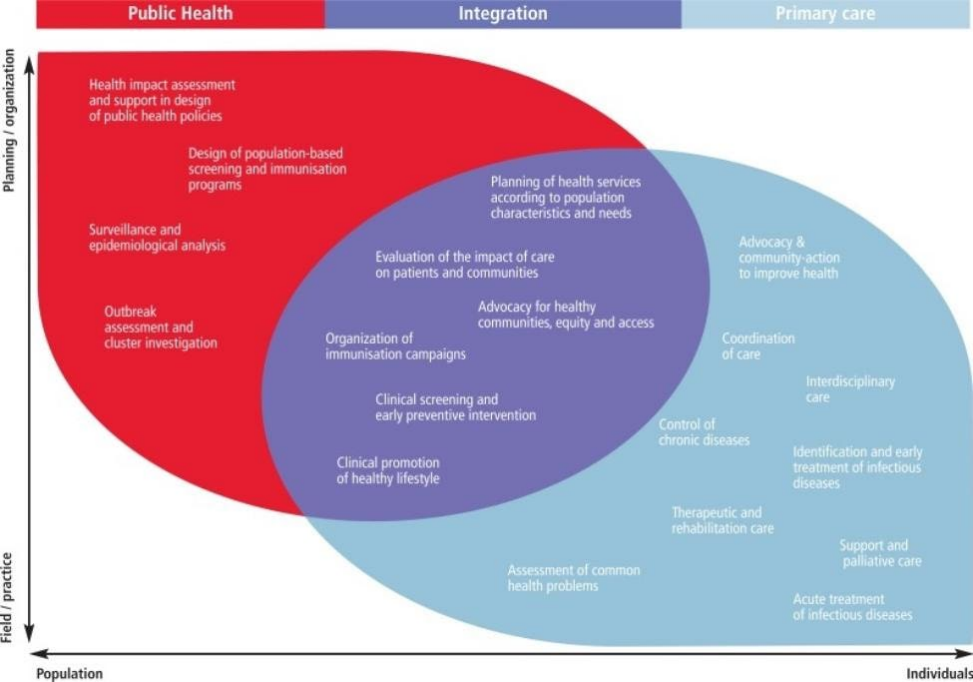
Interaction between public health and primary health care. Source: Levesque et al., 2013 [20]

The structural organization, funding, and delivery of PHC and PH services vary across countries, with some nations maintaining separate systems for these domains, while others integrate them more comprehensively [19]. The appropriateness of each setting largely depends on the specific national context [14]. However, in most countries, PHC performs some PH functions, while PH can help to make the provision of PHC more effective [19]. The complex interplay between PH and PHC was assessed in a review by Levesque et al. [20] and is depicted in Fig. 1. The diagram illustrates that certain functions are distinctly situated within either the PH or PHC domain, while others have overlapping ownership. For instance, screening, immunization, and interventions promoting healthy lifestyles are PH functions that are increasingly being delivered within PHC settings. Conversely, surveillance, planning, and evaluation activities are PH endeavors that contribute to enhancing PHC provision [20, 21]. Both approaches are necessary, and the more closely they are interconnected, the more integrated the services become.

Theoretically, the integration of PH and PHC can be categorized into five distinct areas, which are based on Lasker’s models of Medicine and Public Health Collaborations [22] and the adaptation of these models by Shahzad et al. [23]. This is presented in Table 1.

**Table 1:** Five areas of public health and primary health care integration, based on Lasker’s models of Medicine and Public Health Collaborations [22] and the adaptation of these models by Shahzad et al. [23]

Area of PH-PHC integration	Example
(1) Coordinating health care services for individuals	e.g. by bringing clinical and PH professionals together at one site
(2) Applying a population perspective to clinical practice	e.g. by using population-based information to enhance clinical decision-making
(3) Identifying and addressing community health problems	e.g. by using clinical opportunities to identify and address underlying causes of health problems
(4) Strengthening health promotion and disease prevention	e.g. through education, advocacy for health-related laws or regulations
(5) Collaborating around policy, training and research	e.g. by engaging in cross-sectoral education and training, or conducting cross-sectoral research

The level of integration between PHC and PH can be conceptualized as a spectrum, ranging from isolation to mutual awareness, cooperation, collaboration, partnership, and ultimately, merger [24]. However, it is crucial to emphasize that these terms lack a universally accepted definition and their usage varies considerably in the literature, potentially differing between the North American and European contexts.

Before the COVID-19 pandemic, health care systems differed in their collaborative intersections between PHC and PH. Despite this difference, globally, PHC has been viewed as the front-line responder to the COVID-19 pandemic with the essential role of preventing, preparing, responding to and recovering from an emergency [25]. However, faced with the rapidly evolving pandemic and the lack of protective means and diagnostic tests, most countries initially responded by combining population health management strategies, like introducing containment measures, and with increased acute hospital care [26]. Gradually, PHC sectors have been mobilized to participate in the diagnosis of COVID-19 patients, care for infected individuals with uncomplicated symptomatology, long-term follow-up of complications and vaccination campaigns while ensuring access and continuity of care for all [10, 27]. In many jurisdictions, PHC played an important role in responding to the COVID-19 pandemic, although the specific tasks and the degree of collaboration with PH varied within and between countries [28].

One key area where this collaboration between PH and PHC has been particularly important – especially during the COVID-19 pandemic - was in vulnerable populations. Enhancing the accessibility of PHC services for these populations holds utmost importance in striving for health equity, aiming to align the provision of services with the specific levels of need within communities. Therefore, enhancing collaboration between PH and PHC to ensure that these vulnerable populations receive the care they need is crucial, particularly as the pandemic has disproportionately affected these groups and in it lies the potential to address many of the inequalities that affect health outcomes. Yet, little is known on where the points of collaboration are between PH and PHC to address the needs of vulnerable populations during these challenging times. Furthermore, there exist no frameworks for assessing the impact of PH-PHC collaboration on health equity. Therefore, this paper aims to examine the intersections between PH and PHC serving as a strategy to reduce the impact of the pandemic on vulnerable populations. Also, we assess where these intersections could possibly have an effect on health equity. In this study, we analyze peer-reviewed literature, reports and media articles to develop descriptions of eight jurisdictions to characterize PHC and PH collaboration focusing on vulnerable populations during the COVID-19 pandemic. To identify dimensions of collaboration we apply the World Health Organization’s five key aspects of COVID-19 control: prevention, control of cases and clusters, suppression of community transmission, appropriate clinical triage and care, and vaccination implementation [29]. Next, we examine these jurisdictions to synthesize good practices and enablers of successful collaboration addressing these vulnerable groups, and where these could have an impact on health equity. Finally, we identify opportunities for collaboration between PHC and PH as a strategy to reduce inequity and to prevent inequity gaps to become larger. To date, no study has analyzed the role of PHC collaboration with PH in more than one country, nor examined lessons to be learned across jurisdictions. Our findings will create the foundation for future PHC and PH collaboration studies to address vulnerable populations and to mitigate health inequities during future PH emergencies.

## Materials and methods

### Study design

We used aggregated data from jurisdictional reports from eight countries to obtain information on PHC and PH performance during the COVID-19 pandemic. The reports were analyzed using a framework adapted from the WHO framework for COVID-19 control to identify good practices and enablers of successful collaboration between PHC and PH addressing these vulnerable groups on the one hand and to identify opportunities for collaboration between PHC and PH as a strategy to reduce inequity and to prevent inequity gaps to become larger on the other hand [29]. In this study eight jurisdictions were purposively selected. We looked for jurisdictions with known collaboration between PHC and PH and fulfilling two criteria: (1) having a health insurance scheme with universal coverage; and (2) universal access to PC. This study was limited to high-income countries. The countries included were Belgium, Canada (Ontario), Germany, Italy, Japan, the Netherlands, Norway, and Spain. These countries evolved from a purposive sample of all countries meeting the inclusion criteria. Due to the highly decentralized federated organization of Canada’s health system, we chose one Canadian province (Ontario) as the unit of analysis. Included countries are representative of geographic diversity, health system variability, and jurisdictional policy responses during the COVID-19 pandemic.

Vulnerable populations were defined in this study by the research team as groups of people and/or patients who are vulnerable from a social, financial, or health perspective. People living in vulnerable conditions include individuals who face systemic exclusion and discrimination based on their age, disability, race, ethnicity, gender, income level, religion, caste or creed, gender identity, sexual orientation, and migratory status, in addition to individuals who are caught up in conflict and are stateless, populations who are incarcerated, individuals with chronic health conditions (e.g., mental illness), people living in inadequate housing, and people who are exposed to environmental degradation, air pollution, and at risk due to climate change [2]. Applying this definition of vulnerable populations to the COVID-19 context, this study focused on frail elderly, those with low health literacy and language barriers, people living in a precarious social context, refugees and undocumented migrants, and homeless people as the vulnerable populations at increased risk for adverse health outcomes during the COVID-19 pandemic. For each participating country, the proportion and the composition of these different vulnerable groups are different.

### Recruitment of participants, data collection, and data analysis

First, we identified at least one country expert for each jurisdiction who acted as a co-investigator. These country experts are, due to their experience, known with the PHC field and are also familiar with PH research (see supplementary file for more details on the country experts).

Secondly, a corpus of evidence was assembled for each jurisdiction. To do so a standard report template was utilized, including the following sections: (1) jurisdiction sociodemographic and health system indicators; (2) COVID-19 pandemic progression indicators; (3) the WHO framework [29] for COVID-19 control (consisting of the following 5 topics: prevention of COVID-19 cases, control of cases and clusters of COVID-19, suppression of community transmission of COVID-19, appropriate clinical triage and clinical care for cases of COVID-19, and COVID-19 vaccination implementation); (4) open-ended questions regarding vulnerable populations; and (5) a Strengths, Weaknesses, Opportunities, and Threats (SWOT) analysis. The country experts identified grey literature (e.g., policies, reports, guidelines, media reports and mandates) that describe the role of PHC during the COVID-19 pandemic. Relevant published material was extracted from PubMed, Embase, and Google, as well as the websites of jurisdictional health departments, such as the ministries of health disease control. The country experts searched published literature from March 2020 to August 2021. Each country applied the following standard search strategy including Boolean phrases: (country name AND ‘COVID-19’ AND ‘public health’ AND ‘primary health care’ OR ‘general practice’). This was followed by a snowball technique to identify additional relevant publications. To identify relevant grey literature, the country experts searched for policy reports, policy guidelines and media articles describing the interplay between PH guidelines and the impact on PHC services.

Thirdly, we confirmed these findings with at least five key informants in each jurisdiction who are familiar with PHC and PH within that jurisdiction. We provided them with the draft report from the country expert and asked for feedback, comments and amendments. We also asked the key informants to evaluate the list of used sources and to identify any additional relevant reports or materials.

We provided a narrative synthesis of the extent to which selected jurisdictions address the degree of PHC and PH collaboration as per our framework and added this to the country report. All members of the research team reviewed the final jurisdictional reports, followed by a group discussion. The following questions were used to guide the individual analysis and discussions: (1) What were the different elements of the roles that PHC played in the COVID-19 pandemic response addressing inequity?; (2) Are there common ways of PHC-PH collaboration across jurisdictions to address vulnerable populations?; (3) What were common strengths and weaknesses of PHC-PH collaboration focusing on inequity during COVID-19?; (4) What were common opportunities and threats to reduce inequity gaps identified for future PH crises?; and (5) Which good practices of high-performing PHC-PH collaboration to address vulnerable populations and reduce inequity could be identified?. The research team met virtually to discuss findings and compare results across jurisdictions. To date, the scientific literature lacks established frameworks that provide empirical support for evaluating the influence of PH-PHC collaboration on health equity. However, the subsequent sections present findings that have been highlighted due to the international research team’s consensus hypothesis regarding their potential impact on reducing inequities.

### Ethical considerations

Research Ethics Board approval for this study was not required, as it was using public info and review of documents.

## Results

### Study participants

Table 2 briefly summarizes the characteristics of the health care system in each of the eight jurisdictions under study.

**Table 2:** Health system indicators for each jurisdiction

Jurisdiction	Population size (million)	Regionalization	Type of HC system*	Gini index**	Patient list	PHC payment system	Out-of-pocket payment (% of total CHE***)	PHC practice mode
Belgium	11.5 M	Three regions: Brussels, Flanders, Wallonia	ESHI	27.2	No	Predominantly fee-for-service	18.03	Majority mono-professional group practices.
Ontario, Canada	13.6 M	Ten provinces and three territories	NHI	33.3	No	Mix of PHC payment models: predominantly fee-for-service, blended capitation models, alternate payment models	14.91	Majority group practices
Germany	82.9 M	16 federated states	SHI	31.9	No	Predominantly fee-for-service with quarterly capitation fee	13.12	Majority mono-professional group practices
Italy	60.3 M	107 provinces in 21 regions	NHS	35.9	Yes	Capitation	21.76	Majority mono-professional group practices
Japan	125.6 M	47 prefectures	SHI	32.9	No	Fee-for-service	11.81	Majority mono-professional group practices
The Netherlands	17.2 M	12 provinces	ESHI	28.1	Yes	Combination per practice of fee-for-service, capitation, and pay for performance	11.53	Majority mono-professional group practices
Norway	5.5 M	11 counties	NHS	27.6	Yes	Combination of fee-for-service and capitation	14.59	Majority mono-professional group practices
Spain	46.7 M	17 autonomous regions divided into 50 provinces	NHS	34.7	Yes	Capitation	23.98	Majority multi-professional group practices

*Types of healthcare systems (The definitions underneath are adapted from Böhm et al. [30]*)*:

*National Health Service type (NHS)*: *Regulation, financing and provision are governed by the state* [30]

*National Health Insurance type (NHI)*: *Regulation by the state, financing by taxes, dominantly private service provision* [30]

*Social Health Insurance type (SHI)*: *A dominant role of societal actors in regulation and financing, whereas private for-profit providers mainly deliver services* [30]

*Etatist Social Health Insurance (ESHI)*: *Completely mixed healthcare: state responsible for regulating the system, financing is organized by societal actors, and provision has been delegated to private hands* [30]

**Gini index (as defined by World Bank [31]: *measures the extent to which the distribution of income (or, in some cases, consumption expenditure) among individuals or households within an economy deviates from a perfectly equal distribution. A Lorenz curve plots the cumulative percentages of total income received against the cumulative number of recipients, starting with the poorest individual or household. The Gini index measures the area between the Lorenz curve and a hypothetical line of absolute equality, expressed as a percentage of the maximum area under the line. Thus a Gini index of 0 represents perfect equality, while an index of 100 implies perfect inequality* [31]

***CHE: *Country Health Expenditure*

### Outcomes

Common successes and challenges were found across jurisdictions. We looked at relevant strategies that may affect vulnerable populations positively or negatively, described these strategies in the different countries and highlighted examples of cooperation between PH and PHC, whenever present.

1. Outreach strategies to vulnerable groups.

A recurring theme that consistently emerged from each jurisdictional report was the documentation of outreach initiatives specifically designed to target individuals who encounter barriers in accessing healthcare or are at risk of social isolation. While these initiatives were primarily developed at the local or regional level, they serve as noteworthy illustrations of how PH and PHC efforts aimed to address the needs of vulnerable populations, with the intention of mitigating the widening gaps in healthcare access and health outcomes. However, it is important to highlight that explicit collaboration between PH and PHC was rarely evident in these outreach strategies. Nevertheless, several valuable initiatives were identified that aimed to reach out to vulnerable populations. The subsequent section provides a comprehensive overview of the outcomes pertaining to the various outreach initiatives in each jurisdiction.

Community health workers/ambassadors/stewards/coaches to assist people who needed extra support in testing and quarantining/isolation were present in some countries. For example, in some Belgian municipalities, corona coaches (who could be contacted by PHC professionals) were active in offering help during a period of quarantine or isolation. The coach kept in touch with infected individuals during quarantine and helped if problems were encountered. As such, these coaches provided correct answers to questions about a quarantine or isolation period, assisted in practical matters during a quarantine or isolation period (e.g. doing groceries or walking the dog), and provided a listening ear and mental support. In Spain and Belgium, community health workers (CHWs) were involved with vulnerable communities and stayed in contact with vulnerable individuals experiencing social isolation while offering them support from the community [32]. In the Netherlands, GPs were advised by their professional association to contact the Red Cross, which had trained volunteers available to support vulnerable people in isolation, for instance, by providing help with shopping or listening to the worries of people. In Norway, an “Ambassador” project was launched to reach Somali immigrants during the pandemic, which failed in many of its attempts at the start. Still, it gradually became more successful as the experience was gained during the project. Particularly, it was necessary that outreach personnel not only had language skills but also possessed high cultural competence and legitimacy in the target group [33]. The St. Michael’s Hospital Academic Family Health Team in Canada executed wellness check-ins by means of electronic medical record (EMR) searches in order to detect the health and social necessities of their patients. During the period from March to August 2020, nurses, physicians, and other personnel conducted more than 2000 wellness checks, thereby identifying the health and social needs of patients and connecting them to team and community resources [34].

In Japan, social prescribing has been identified as a crucial tool for addressing the lack of social connections and interactions, particularly in the context of the COVID-19 pandemic. Social prescribing is a method of addressing social issues and promoting community wellbeing by providing non-medical solutions such as social activities, community involvement, and other forms of social support. One social prescription that has been used in Japan to address social isolation is the Osekkai conference [35]. The Osekkai conference is a community-based initiative that aims to promote social participation and interaction, particularly in rural communities. The conference brings together community members to discuss and address local issues, share information, and build social connections. By fostering social interactions among conference participants, the Osekkai conference has the potential to reduce loneliness and improve overall community wellbeing. Overall, the Osekkai conference is an example of how social prescribing can be used to address social isolation and promote community wellbeing in Japan, particularly in the context of the COVID-19 pandemic [35].

In Italy, mobile primary care teams called USCA (Unità Speciale di Continuità Assistenziale: Special Continuity Care Unit) were implemented as an outreach service (although not specifically oriented to vulnerable groups) involved in telephone triage, assessment and home care [36]. The USCA service represented the operational extension of GPs in the fight against COVID-19 and in the provision of support at home to vulnerable COVID-19 patients. The referring GP initiated contact with the USCA service, which then evaluated the case through an assessment conducted by a USCA doctor. The severity of a case determined whether the USCA doctor arranged a home visit, providing prompt hospital referral if indicated for patients suspected or confirmed to have COVID-19. In the initial phase of the pandemic, there was a notable increase in the number of cases presented to emergency rooms at very late stages of the illness and often in a compromised state. This trend was attributed to the population’s fear of hospitalization and the difficulties associated with obtaining appropriate care in the community. Consequently, the introduction of the USCA service represented a significant improvement in the management of COVID-19 cases outside of hospital, within the community setting.

In certain instances, mobile teams were deployed as an outreach strategy to conduct testing and administer vaccinations to vulnerable groups, particularly homeless individuals and refugees. For instance, in the Netherlands, PH services utilized vaccination buses to visit regions with low vaccination coverage, providing individuals the opportunity to receive vaccinations without prior appointments. Similarly, in Italy, there were instances of mobile vaccination clinics established to reach areas with low vaccination rates and populations classified as “hard to reach.“.

Additionally, isolation centers for homeless people or refugees living in congregate settings evolved. A main challenge for this vulnerable group arose as general measures to stay at home became a standard during the first lockdown period. In Belgium, an inter-federal Task Force on Vulnerable Groups has been established, with the aim of providing resources to expand the range of shelters available to homeless people [37]. Moreover, various humanitarian volunteer initiatives have been established by non-governmental organizations (NGOs including Doctors without Borders, Doctors of the World, Red Cross, etc.) to address the needs of these marginalized populations. These initiatives involved the creation of mobile teams, information centers, testing facilities, medical care centers, and isolation facilities, among other things [38]. In Toronto, Inner City Health Associates – a primary care organization that serves 70 + shelters in Toronto – pivoted to provide care to people experiencing homelessness in temporary housing, often in vacant hotels [39, 40]. Also in Italy, a system of regular medical care was established at COVID-19 hotels. At the start of the pandemic, several tourist hotels were repurposed to serve as COVID-19 hotels, with the purpose of accommodating individuals facing housing challenges, socially vulnerable patients, and those requiring quarantine after traveling from high-risk countries. Typically, the COVID-19 hotels were staffed by nurses, with USCA doctors available to provide assistance as needed [36]. In Germany, however, rather than actively outreaching through mobile teams and expanding the capacity for shelters, the opposite could be noted: there is evidence that shelters had to reduce their capacity and operating hours [41]. Services were limited for this homeless population, for whom a rise in mental instability was noted. The health authorities did assume the responsibility of institutions in providing care for homeless people and were insufficiently engaged in taking over the organizational effort of caring for this group [41].

A summary of these results is provided in Table 3.

**Table 3:** Examples of initiatives for outreach strategies to vulnerable groups during the COVID-19 pandemic for each jurisdiction

Jurisdiction	Examples of initiatives for outreach strategies to vulnerable groups		
	**Medical care**	**Social care**	Availability of hotels/shelters*
Belgium	NGOs (Doctors without Borders, Doctors of the World, Red Cross, etc.)	Corona coaches CHWs	Yes, through an inter-federal Task Force on Vulnerable Groups
Ontario, Canada	Care as usual	Wellness check-ins	Yes, Inner City Health Associates turned vacant hotels into shelters for homeless people
Germany	Care as usual	Not documented	Yes, but less places than expected
Italy	(USCA: GPs, although not specific to vulnerable groups)	Not documented	Yes, COVID-19 hotels
Japan	Care as usual	Social prescribing; e.g. Osekkai conference	No
The Netherlands	Care as usual	NGOs (Red Cross volunteers) s	Yes Vaccination busses of the PH services went to areas with low vaccination coverage
Norway	Care as usual	Ambassador Project	No, because only a very small population of homeless and refugees
Spain	Care as usual	CHWs	Yes

*Availability of hotels and/or shelters for isolation for COVID-19 patients, quarantine for COVID-19 contacts and for people who need special care or who lack a place to stay

2. Digital solutions for distanced care.

In each of the countries involved in the study, the implementation or expansion of remote patient assessments through telephone, email, and video consultations was observed. In order to address the imperative of reducing COVID-19 transmission within healthcare settings, legislative measures and practice guidelines were frequently modified. For example, the requirement for in-person visits to obtain sickness certificates was eliminated in some settings. Also, policies such as reimbursement codes for virtual or telephone consultations were implemented to overcome the obstacles associated with telehealth. This emphasis on remote alternatives for consultations was consistent across all jurisdictions. However, it is important to note that these digital alternatives do not represent a collaborative effort between PH and PHC specifically targeting vulnerable populations. Rather, they serve as an example of policy developments aimed at improving access to PHC services and reducing the burden on PHC professionals, particularly GPs. We include this information in our report because it has the potential to impact access to care and, consequently, equity. However, it is crucial to acknowledge that there is currently insufficient scientific evidence to support these assertions. Additionally, the perceptions regarding these digital solutions varied considerably among the different jurisdictions.

Notably, in the Netherlands and Norway, supplementary financial assistance, including reimbursement for telephone consultations, was expeditiously arranged to support PHC, leading to significant enhancements in direct access to PHC electronic patient records within hospitals. Another example in Germany shows that digitalization processes created new ways of communication in long-term care facilities (LTCFs) between the non-visiting GPs and the caring nurses [42]. In Spain, most of PHC services are provided directly by public PHC practices and no incentives to use remote assessment were provided. In Norway, digital platforms increased the workload of GPs, being accessible 24/7 and possibly with a too low threshold for patients to use it almost as a chat function. Patients could contact the GP with simple questions, or electronic consultations or video consultations, and the former two tend to increase the workload. Moreover, not all GPs had solutions in place for video consultations. In Japan, some online services have accelerated. Yokohama city provided the social-impact bond program offering online medical consultation services for perinatal women for free. Although perinatal women are not acknowledged to be a vulnerable group, they can be considered as vulnerable in the context of the COVID-19 pandemic since they are at increased risk of COVID-19 infection related complications that can affect the pregnancy on one hand and since perinatal women are at increased risk of anxiety and depression, which may be exacerbated due to the lockdown and distancing measures on the other hand [43]. This particular case serves as an illustration of how a specific subgroup of patients, experiencing a distinct stage in their lifespan with increased risks, may necessitate heightened medical attention, which could potentially be compromised due to containment measures. By addressing their access to information, we highlight the potential benefits of digital solutions in improving equitable access to health information. A summary of these results is provided in Table 4.

**Table 4:** Digital solutions during the COVID-19 pandemic for each jurisdiction

Jurisdiction	Digital solutions		
	**Remote assessment**	**Reimbursement for phone consultations**	**Examples of experiences in digital health**
Belgium	Yes	Yes	Online consultations
Ontario, Canada	Yes	Yes	Online consultations
Germany	Yes	Yes	Improving communication with LTCFs
Italy	Yes	No	Online consultations
Japan	Yes	Yes	Online consultation for some groups (perinatal women)
The Netherlands	Yes	No	Online consultations
Norway	Yes	Yes	Online consultations
Spain	Yes	No	Online consultations in some regions

3. Primary health care and public health representation at the decision-making level.

In the majority of the participating countries, authorities placed PHC professionals on the “front line” to play a significant role in containing the pandemic. However, they were generally underrepresented in government expert panels deciding on guidelines and measures, while PH experts were frequently involved in these committees. This mainly accounts for PH experts operating on the national level. In contrast, PH experts working at the local level had in general limited involvement in government expert panels. With the exception of Norway, no PH officers were present at local PHC services in the participating countries. As a result, pandemic plans were primarily developed and shared at the state level, with knowledge disseminated to local PH offices. Consequently, decision-making on guidelines and population measures primarily flowed from PH authorities and specialized care at the national level, rather than from the local level. PHC professionals were predominantly tasked with translating these new guidelines for the population. Notably, strategies aimed at translating guidelines specifically for vulnerable groups were often initiated before the development of government-issued guidelines, if such guidelines were even formulated. This was due to the spontaneous implementation of strategies by organizations already engaged in working with vulnerable populations prior to the pandemic.

This approach had several implications. For instance, the initial identification of patients classified as “clinically extremely vulnerable” for shielding purposes relied mainly on hospital records, assumed to provide the most accurate clinical information, which was later verified by general practitioners (GPs). Consequently, this method resulted in the misclassification of a significant number of patients initially [44]. Furthermore, only a few countries were able to access PC data and link it with other PHC services or hospital data to identify vulnerable patients. Consequently, real-time PC data were largely unavailable, hindering the integration of PHC into the acute phase response. As a consequence, achieving proportionate universalism in policy decision-making and guideline development, tailored to the needs of vulnerable groups and ensuring their acceptance, became challenging to accomplish. Consequently, equitable care and health outcomes were indirectly affected.

4. Need for clear level of engagement and communication channels between public health and primary health care.

In most countries, covered by our study, PHC professionals received news about PH decisions (regarding guidelines and protocols for the safe delivery of care and infection prevention and control during the pandemic) through the media and occasionally through messages from local PH officers or directives from the Ministry of Health. When this communication did occur, it was fairly rudimentary, for example, sending out newsletters about updates. This limited communication was one-way from PH to PHC professionals. Mostly, there was no significant effort to build a PHC advisory table, where the broader actors of PHC could be informed. In some places, PHC professionals and specifically GPs self-organized to transmit PH information and adapt to local contexts. For example, the Ontario College of Family Physicians in Canada co-organized bi-weekly webinars with the University of Toronto Department of Family and Community Medicine (DFCM) to ensure timely and accurate COVID-19 information was transmitted to GPs [45]. Some webinars had over 1000 GPs joining for the session.

Where communication channels and information on decision making mostly worked in a one-way direction, there was a minority of countries that mentioned on initiatives to feedback information from the frontlines to the government. For example, in Germany and Belgium, local coordinating doctors were appointed, mostly per municipality, to participate in a task force aiming as a sort of front-line feedback system to pick up signals and experiences from their communities. These forms of population surveillance and management applied to the general population as a starting point and could focus on vulnerable populations if specific issues arose. Also, in Belgium, population managers operated at the local level for each Primary Care Zone and played a connecting role between vulnerable populations and PHC actors. They spoke to partners in the field who are in contact with these vulnerable groups to organize and adapt, for instance, the vaccination program.

## Discussion

This paper examines the points of intersection between PH and PHC to reduce the impact of the COVID-19 pandemic on vulnerable populations. To date, no other study analyzed the role of PHC-PH collaboration in multiple countries, nor identified lessons to be learned across jurisdictions to reduce inequity gaps. Four thematic axes emerge from the analysis: outreach strategies to vulnerable groups, digital solutions for distanced care, PHC representation at the decision-making level and the need for clear communication channels. These four themes evolved from the international research team’s consensus hypothesis regarding their potential impact on reducing inequities, since the scientific literature lacks established frameworks that provide empirical support for evaluating the influence of PH-PHC collaboration on health equity. In general, some cross-jurisdictional conclusions can be made.

First, sustaining access to PHC and the continual management of all health-related issues is important and should target vulnerable populations as well. A major strength of PHC practices is their ability to respond to local needs. Nevetheless, our findings show the importance of having CHWs or NGOs to understand the difficulties experienced by vulnerable populations to access the healthcare system on a local level. Furthermore, enhancing accessibility to PHC necessitates an intersectional approach involving various stakeholders, such as public health entities, PHC providers, and community players like CHWs in order to effectively tackle this issue. The response to COVID-19, both at the individual and organizational levels, has been swift and remarkable. Within this response, there were valuable initiatives to try to reach out to vulnerable populations. Nevertheless, they were hardly any examples of explicit collaboration between PH and PHC and a comprehensive and coherent strategy has been lacking. PHC presents opportunities for early intervention to mitigate adverse outcomes, to provide sustained care, and improved overall system resilience in response to the various health challenges that arise from the continually evolving COVID-19 pandemic. The effectiveness of technical and human responses necessitates the direct participation of PHC and PH in the formulation and execution of service adjustments and the provision of appropriate training, all of which should be founded on sound research evidence [44].

Secondly, digital solutions to the health crisis led to high uptake of virtual care but could also have been a barrier for vulnerable populations in accessing PHC. Globally, evidence to date notes high uptake of virtual care; in fact, most patients report being able to access care [2]. Although this digital switch can solve low access to care for some vulnerable groups, of course, not all vulnerable groups, among others elderly, will be reached this way. It is necessary to ensure that endeavors aimed at encouraging the use of online tools to access crucial information are equitable and facilitate continuity of care [44]. A study in Ontario on healthcare access demonstrated that digital solutions helped alleviate the problem of access to care during the pandemic; however, individuals who were employed tended to opt for scheduling phone or online consultations with their PHC provider. This has implications for those not currently working or whose work requires them to be on-site and physical, with more barriers to initiating contact with their PHC provider [46]. Also, the lack of prior education and training of PHC professionals with regard to digital skills might pose challenges to the implementation of digital solutions in supporting a PH response. Furthermore, despite its usefulness, telemedicine has limitations; physical touch is a critical component of patient evaluation and care, and patients’ suitability for online consultations varies. The pandemic has pushed the era of the internet forward, yet nearly half of the world’s population still lacks connectivity. Although these digital alternatives do not represent a collaborative effort between PH and PHC specifically targeting vulnerable populations, they do serve as an example of policy developments aimed at improving access to PHC services, thereby potentially also having an impact on equity. The COVID-19 pandemic emphasized the digital divide’s profound inequity during periods of lockdown. As a result, the capability to introduce new care approaches must be evaluated and, when appropriate, sustained as positive indications of PHC’s responsiveness [10].

Thirdly, the pandemic has revealed the complex interconnection among different types of inequities that existed and were expanding in and between nations even before the pandemic [47]. By analyzing intersections and their interaction with health and socioeconomic disparities, the global health community is now better equipped to design health and social care interventions that are context-specific and community-based, as well as tailored to national-level needs [2]. However, evidence indicates that in many places PHC services, including civil society and community organizations, serving these populations have had little impact on government decision-making [48]. Consequently, strategies aimed at vulnerable groups were mostly initiated by civil society, prior to the development of specific policy guidelines – if those were developed- and on a temporary basis. This was also confirmed in this study, as guidelines concerning outbreak control and strategies for health promotion were, in general, developed by government expert panels, in which PHC professionals were mostly not represented, nor were the vulnerable communities themselves. Moreover, it is very different if PHC professionals or vulnerable communities are represented, as the first ones not necessarily are aware of or represent the needs of such communities. As such, both PHC professionals, who are aware of problems posed by providing services to vulnerable groups, and vulnerable communities themselves were not represented in expert panels at an institutional level. There is a clear disconnect between the ‘invisibility’ of certain groups at the institutional level (as evident from the absence of PHC data concerning vulnerable populations), and the actions undertaken by a number of informal and – often – also formal actors within the gaps that are not adequately addressed by structured interventions and policies. Nevertheless, involving PHC professionals and vulnerable communities in policy-making decisions is essential because they can propose solutions, promote adherence, and customize responses to address the unique and varied needs of different populations [2, 49].

Fourthly, at the country level clear communication channels should exist that can be rolled out at the local level and can be adapted to local needs. However, there was little shared knowledge and no existing structures to support this. As a consequence, PH in general remains less visible to PHC at the local level and vice versa. It is still hard to reach PH through PHC providers. However, during and after the pandemic, PHC professionals could have a crucial role to play by leveraging their information infrastructure to identify high-risk groups, track adherence to guidelines, offer tailored care, and detect novel cases of COVID-19 infection [10]. At the local level, some PHC practices did create personal initiatives to prevent problems in their vulnerable patient populations, but a broader population perspective was lacking. Also, many PHC professionals do not habitually take on a PH perspective; initiatives to pick up signals from vulnerable communities and to develop interventions depend on how the PHC professional sees his/her role in this. Some practices may have had a pro-active attitude, but generally, PHC professionals work more on an on-demand basis: the patient initiates the contact with the PHC professionals, and the EMR is then consulted. To take on this broader population perspective, PHC professionals need tools to use the EMR as a strategy for PH initiatives and channels to communicate with PH levels. This is also confirmed by the fact that the lack of real-time PHC data has hampered the positioning of PHC in acute phase response. This communication gap between PH and PHC existed before but has been exposed by the COVID-19 pandemic. The gap points to persistent underfunding of the PH system – less than 5% of all healthcare budgets in all countries included in this study – and how PH cannot engage with every PHC professional in their area. While PH is to some extent integrated with local government, it remains largely disconnected from PHC and vulnerable populations. This disconnection has led to inadequate health protection measures in numerous residential care homes, among other examples. Also, the consequences for mental wellbeing as a result of the isolation measures for vulnerable groups were insufficiently anticipated, as counts for the initial underestimation of the impact on people living in LTCFs. This problem is exacerbated by the fact that PH was initially marginalized in testing and contact tracing efforts, which were outsourced to private companies instead of reinforcing existing public services. This has had a detrimental effect on the coherence of the overall response to the situation [44]. Overall, the absence of effective communication channels between PH authorities, PHC professionals, and vulnerable communities undermines efforts to address health equity by impeding understanding, access to information, alignment of interventions, and meaningful participation. Establishing robust and inclusive communication channels is crucial for ensuring that healthcare services and policies are responsive, equitable, and effectively address the health needs of all individuals, particularly those who are vulnerable.

To conclude, the response to the COVID-19 pandemic has highlighted the crucial role of an integrated PHC system. The effectiveness of the demanding work of PHC professionals, such as GPs, nurses, social services and community organizations, depends on their integration with other key actors, including PH experts. The concept of population health is increasingly being employed to establish connections between healthcare services, PH agencies, social services, and behavioral health services, as well as other stakeholders including employers and schools, with the goal of enhancing health outcomes within communities [26]. Thus, pandemic response and planning ought to take into account the social determinants of health in order to mitigate the disproportionate effects of COVID-19. In order to comprehend the precise impacts of COVID-19 on vulnerable populations, it is necessary to conduct data collection that is more current and comprehensive, focusing on hospitalization and mortality rates, as well as other health, social and wellbeing measures, which are stratified by income, gender, age, race, ethnicity, disability, and other pertinent variables [2]. Consequently, health policy interventions must consider the heightened vulnerability associated with social determinants of health and unequal access to healthcare. The findings in this study show that the current collaboration between PH and PHC still needs to be improved to establish strong and coordinated population health management strategies with special attention for a proportionate universalism approach in order to reach the most vulnerable ones in the population. PH professionals have a crucial role to play in resolving health disparities and addressing social determinants of health, with the potential to effect change at the individual, practice, and community levels [50]. At the individual level, they may engage in conversations with patients regarding potential social barriers to optimal health outcomes. At the organizational or practice level, they can identify strategies to mitigate difficulties in healthcare access. Finally, at the community level, they may collaborate with community-based organizations to address social determinants of health and promote health equity [1, 50]. In addition, PHC professionals are an integral component of a cohesive strategy aimed at strengthening the health system’s capacity to respond to COVID-19 outbreaks, concurrent epidemics, and the postponed management of other illnesses. Timely communication and collaboration among PHC and PH are crucial to this end. The competence of PHC in identifying vulnerable populations and the proficiency of PH in effectively reaching out to them underscore the potential benefits of closer collaboration. Hence, fostering greater cooperation between these entities is expected to reinforce the strategies aimed at addressing the distinctive needs of vulnerable populations. In order to achieve the goal of equitable health provision, address the underlying determinants of health, and mitigate the effects of the inverse care law, it is necessary to establish regular, close, and horizontal collaborations among all stakeholders involved in the complex care process. Also, it is important to address macro-level barriers to this part, such as the scarce availability of real-time PHC data on vulnerable populations hampering the position of PHC in the acute phase response, the differences in governance structures posing challenges to collaboration and the lack of prior education and training of PHC professionals posing challenges to the implementation of digital solutions in supporting the PH response.

### Implications for practice and research

This paper formulates recommendations for government actors and care providers, both at a national and local level to consider when developing short-, medium- and long-term strategies to address vulnerable populations and to prevent inequity gaps to become larger during times of health crisis. Pandemic preparedness plans and other crisis management protocols should incorporate enhanced definition and protection measures for vulnerable groups, including the training of PH professionals to do so [51]. Also, more research into PHC-PH collaboration during a pandemic is needed. The COVID-19 pandemic presents opportunities to assess the efficacy of early clinical interventions and to swiftly implement potential long-term modifications in healthcare services. Thereby, the thorough evaluation of near-patient technologies in community settings is imperative to ascertain their safety and efficacy [44]. At this critical juncture, it is necessary for all sectors to work collaboratively and cooperatively to narrow inequity gaps, enhance universal health coverage and social protection, and implement a health-in-all-policies approach [2].

### Limitations

There are some limitations to this study. First, the countries which participated in reporting were self-selected, and though they represented a diverse geography and set of cultures, the experiences and lessons from these countries are limited and cannot be generalized to all high-income countries. Also, this cross-jurisdictional analysis is limited by each jurisdiction’s unique history and evolution of PHC and PH systems. Second, data collection relied on published literature, reports and media, followed by a confirmation with stakeholders. This synthesis, although performed by investigators with a professional background in PHC, may only partially capture some collaboration initiatives between PH and PC that occurred. Each co-investigator only knows a certain amount about what happened in their own country. Furthermore, regional differences between countries can still occur. Many details may only be uncovered through interviews; this could be a direction for future research.

## Conclusions

This study identified opportunities for collaboration between PHC and PH to reduce inequity gaps and to improve population health, focusing on vulnerable populations. Careful, intentional coordination between the two systems should be established in normal times as a basis for effective response during future public health emergencies. The pandemic has provided significant insights on how to strengthen health systems and provide universal access to healthcare by fostering stronger connections between PH and PHC.

### Electronic supplementary material

Below is the link to the electronic supplementary material.

**Additional file 1:** Country experts

## Data Availability

The dataset used and analyzed during the current study are available from the corresponding author on reasonable request. All data are centrally stored on the Ghent University server (Belgium).

## List of abbreviations

CHW: Community health worker
GP: General practitioner
NGO: Non-governmental organization
PHC: Primary health care
PH: Public health
LTCF: Long-term care facility
