# Supplementary material for: Addressing health inequity during the COVID-19 pandemic through primary health care and public health collaboration: a multiple case study analysis in eight high-income countries
Source: Int J Equity Health. 2023 Aug 31;22:171. doi: 10.1186/s12939-023-01968-6 (PMC10472729; doi:10.1186/s12939-023-01968-6)
Supplement: Supplementary file 1 — Additional file 1: Country experts [file 12939_2023_1968_MOESM1_ESM.docx]

# Supplementary: Country experts

Dorien Vanden Bossche MD PhD(c) is a general practitioner and PhD candidate at Ghent University Department of General Practice and Primary Health in Belgium.

Q. Jane Zhao is a Health Services Research PhD student at the University of Toronto’s Institute of Health Policy, Management and Evaluation (IHPME) in Canada.

Carnelle Lawes MPH(c) is an MPH candidate in the Faculty of Health, Master of Public Health at the University of Waterloo and a trainee at the Upstream Lab in Toronto, Canada.

Maria Pilar Astier Peña MD MBA PhD is a general practitioner and quality officer in Territorial Healthcare Quality Unit of Camp de Tarragona. Healthcare Institute of Catalonia (Spain) and Patient Safety Working Party chair of semFYC, Madrid, Spain.

Sara Ares Blanco MD is a general practitioner in Healthcare Service of Madrid Region (Spain) and semFYC representative at the European General Practice Research Network in Madrid, Spain.

Guri Rortveit MD PhD is general practitioner and Head of the Department of Global Public Health and Primary Care at the University of Bergen in Norway.

Madelon Kroneman PhD is a senior researcher at Nivel (Netherlands Institute of Health Services Research) in Utrecht in the Netherlands.

Emmily Schaubroeck MD is a general practitioner and researcher at the Institute of General Practice, Friedrich-Alexander-University Erlangen-Nürnberg (FAU), Erlangen, Germany.

Stefanie Stark is a sociologist and researcher at the Institute of General Practice, Friedrich-Alexander University Erlangen-Nürnberg (FAU), Erlangen, Germany.

Ferdinando Petrazzuoli MD, PhD has been a general practitioner in Italy for 35 years. He is a post-doctoral researcher at the Center for Primary Health Care Research, Department of Clinical Sciences in Malmo, Lund University, Sweden.

Alice Serafini MD is a general practitioner and researcher at the University of Modena and Reggio Emilia in Italy.

Naoki Kondo MD PhD is a medical doctor and professor in the Department of Social Epidemiology at the University of Kyoto in Japan.

Daisuke Nishioka MD CSW PhD is a medical doctor, social worker and lecturer at the Department of Medical Statistics, Research & Development Center at Osaka Medical and Pharmaceutical University in Japan.
